# Supplementary material for: Mutations in PCYT2 disrupt etherlipid biosynthesis and cause a complex hereditary spastic paraplegia
Source: Brain. 2019 Oct 22;142(11):3382–97. doi: 10.1093/brain/awz291 (PMC6821184; doi:10.1093/brain/awz291)
Supplement: awz291_Supplementary_Data [file awz291_supplementary_data.zip › awz291-Suppl_data/Supplementary_Data4.zip!/Supplementary information 4 - plasma lipidomics.zip!/Plasma lipidomics/Control vs Patient1/Statistical plots and data/SM[t] species/Random forest.pdf]

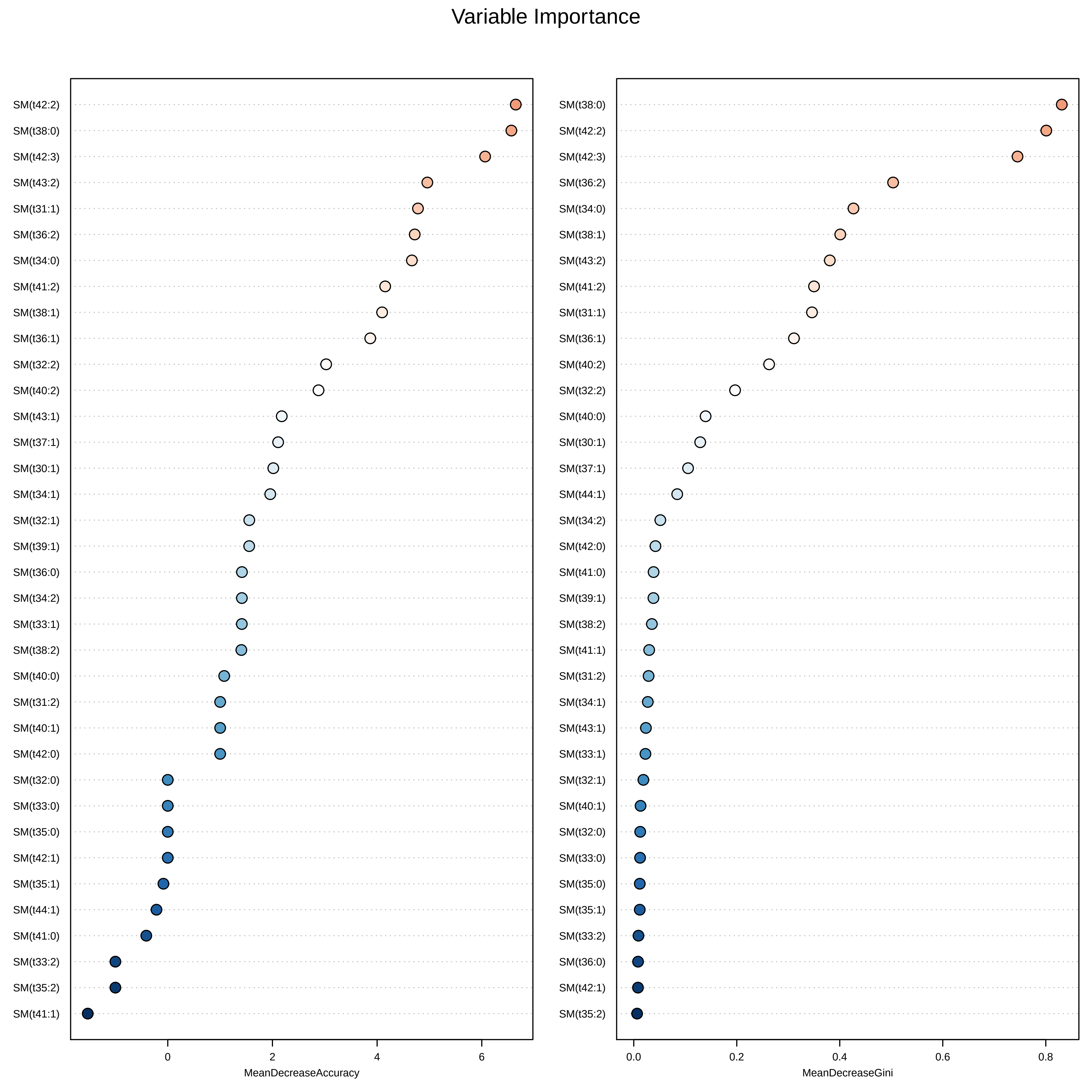
Variable Importance
SM(t42:2)
SM(t38:0)
SM(t42:3)
SM(t43:2)
SM(t31:1)
SM(t36:2)
SM(t34:0)
SM(t41:2)
SM(t38:1)
SM(t36:1)
SM(t32:2)
SM(t40:2)
SM(t43:1)
SM(t37:1)
SM(t30:1)
SM(t34:1)
SM(t32:1)
SM(t39:1)
SM(t36:0)
SM(t34:2)
SM(t33:1)
SM(t38:2)
SM(t40:0)
SM(t31:2)
SM(t40:1)
SM(t42:0)
SM(t32:0)
SM(t33:0)
SM(t35:0)
SM(t42:1)
SM(t35:1)
SM(t44:1)
SM(t41:0)
SM(t33:2)
SM(t35:2)
SM(t41:1)
0
2
4
6
MeanDecreaseAccuracy
SM(t38:0)
SM(t42:2)
SM(t42:3)
SM(t36:2)
SM(t34:0)
SM(t38:1)
SM(t43:2)
SM(t41:2)
SM(t31:1)
SM(t36:1)
SM(t40:2)
SM(t32:2)
SM(t40:0)
SM(t30:1)
SM(t37:1)
SM(t44:1)
SM(t34:2)
SM(t42:0)
SM(t41:0)
SM(t39:1)
SM(t38:2)
SM(t41:1)
SM(t31:2)
SM(t34:1)
SM(t43:1)
SM(t33:1)
SM(t32:1)
SM(t40:1)
SM(t32:0)
SM(t33:0)
SM(t35:0)
SM(t35:1)
SM(t33:2)
SM(t36:0)
SM(t42:1)
SM(t35:2)
0.0
0.2
0.4
0.6
0.8
MeanDecreaseGini
